# Ancient and Modern

**Published:** 1899-07

**Authors:** Charles Fox


### Ancient and Modern.

BY CHARLES FOX, L.D.S.

At the very time the stalwart humanity of England, France and Spain were preparing surgical subjects of one another in Trafalgar Bay, a tiny maiden peeped out into the daylight in an ancient Cotswold town, and heralded her first birthday with a lusty scream. Though well nigh four and ninety years have passed by since then, the same individual hale and hearty, comely and cheery, is welcoming the first signs of spring-time as eagerly as ever. Down the steep street of the little town, between old-fashioned houses with overhanging eaves, which seem to look down with kindred feelings on the bent figure of the ancient lady, she slowly paces by with her stout oaken stick. Many greetings from young and old she exchanges, until a seat at the outskirts is reached and she sits down to look again at the familiar landscape.

It is a splendid view of rolling hills and beechwoods, distant river and valley, that rewards the wayfarer, and no sound but the tinkle of sheep bells, the lark carolling overhead, and the far hum of wheels astir in the human hive below.

She is a grand old woman, this widow of the late Dr. Hinton, renowned a generation ago for his sterling common sense and broad humanity. Her hair is white as snow, but plentiful, and her teeth look strong and capable of many years' service yet. There is no sign of failing in the keen eyes, or trembling in her firm hand crossed by a thousand wrinkles.

But another wayfarer of a very different style is resting at the other end of the seat. She is small, pale and intellectual looking, with gold-rimmed glasses, and prematurely rounded shoulders. There is an unmistakable city air about the young lady, a victim evidently to the higher education of women upon the forcing system. All sorts of unrealities hovered about the modern article, false hair, false teeth, an artificial estimate of life, an atmosphere removed from the wholesome touch of genuine people. While Mrs. Hinton from top to toe looked real and sensible, a breezy hill-top woman, whom any-one might be proud to call grandmother. A listener was a luxury to the old lady, so she opened fire by pointing out a great stone building in course of erection on the opposite hill, and remarking:

"For once the doctors seem to be on the right track, but they are only repeating history with their open-air treatment of consumption."

"Is that Dr. Parr's sanitorium on the new system, for tubercular patients?" said the young lady, after peering across the vale with her short-sighted eyes.

"Not very new, my child, for I remember sixty years ago Dr. Hinton regularly prescribed out-of-door doses for every one tainted in that way. 'Follow the plough,' was his favorite method, and scores of little lads and lassies have I seen come in after trudging miles behind in the furrows, up and down, up and down, all day long. Out again next morning, wet or fine. Whenever it was fit for the men and horses it was fit for the consumptives, so he said. Folks thought it was the newly turned earth that effected the cures, but we knew it was being out in the open air and working up a keen appetite that brought color to the cheeks and breadth to the chest."

"But you would hardly get Dr. Parr's patients to humiliate themselves by tramping after a common ploughman," replied the other with a slight curl of the lip.

"Dr. Hinton was a man of different metal, my dear, and if his patients, rich or poor, rebelled against his orders, he had a habit somehow of managing them for their own good. Instead of building a fine palace like that new-fashioned, windowless affair on the German model, where a few well-to-do, idle invalids will waste the substance of half a township, he would have set each one some useful, interesting task that would have coaxed them into wholesome ways. Nothing is worse in my view than crowding a lot of people together who are all thinking only of their own health. One young curate going off into rapid consumption he persuaded to work in that quarry with crowbar, shovel and pickaxe. The doctor set him the example, and the work soon became fascinating in itself. Why, my child, his sermons were worth ten times as much when he got into touch with the realities of life, and his lungs were so thoroughly rinsed with this hill air that he laughed the consumption all away. But there, none of the young men doctors come up to Dr. Hinton, with all their bacteriology and X rays."

"You may be interested in knowing that my profession rather prides itself on the researches in those fields. I am a dental surgeon," said the young lady.

"Well, to be sure! I suppose the people of to-day want help in that way; though there wasn't even a man dentist in the district forty years ago. And what a shameful set of mouths the young girls have, and how they grin at one another with rows of china-looking things all arranged in regular order! It's well you stopped me in time, for I might have said a word or two about some of our city dentists down yonder, not over polite."

"'Oh wad some power the giftie gie us
To see ourselves as others see us,'"

quotes the youthful L.D.S., almost involuntarily concealing her double row of "china-looking things" from the gaze of this terrible old critic.

"It isn't often I venture so far as the city now, but last week I went with a poor little kitchen maid who had been shut up by some cruel grand folks in a dingy underground scullery. Of course, all her teeth were going to pieces and her face was as pale as paper. There were several miserable mortals waiting in the dentist's room when we got there, but as they seemed to go out in turn I sat and said nothing. But when a pair-horse carriage drove up and an overdressed woman who called herself Lady Blank walked in and told the buttons she must see the dentist at once, I woke up a bit. 'Have you an appointment, madam?' said I. 'No,' she responded shortly. 'Then you ought to be ashamed of yourself to push in front of a suffering child like this. You look strong enough to wait an hour.' And before my lady had recovered herself I marched the maid into the surgery and put her in the big machine chair. Mr. Dentist looked a bit surprised and not over pleased, but I suppose I was rather too old for him to quarrel with. Then they had in another man from next door to give the child gas, and in less than five minutes we were shown out with six teeth gone and a month's wages taken from the kitchen maid's pocket. It may be all right, my dear; perhaps you can explain to me how it is fair for two strong men to exchange five minutes work for a poor girl's four weeks labor; but all I know is Dr. Hinton would have starved before he did like that."

"But you perhaps forget the expenses our city dentists incur before they gain anything for themselves; and, after all, there are hospitals for the poor," said the modern representative.

"I may be wrong, my dear, but it is these very expenses that are the evil, I believe. Dr. Hinton lived simply among his people, poor almost as they. We had no buttons at the door, for as often as not he or I answered the bell ourselves. We could afford to be generous because we spent so little on ourselves and our establishment. Hospitals will never fill the place of kindly teachers of health, who go in and out among the people and show them how to live aright. That is the true office of either dentist or doctor, not to set up gilded traps to catch the rich sick ones, who would be better if they sought for health in a share of honest toil. Nor are they trimming the balance when they hurry through a crowd of cases at a dismal hospital. I am fast nearing my journey's end, young lady, but my sight is clear in many things as I look back over scores of years of work among the sick. We don't want to study disease so much as to keep our eyes fixed on the sun of health. Our doctors and dentists should not waste their time in naming the varieties of microbes, but show the people how to walk in the light so that no evil thing can touch them. If you live to my age you may see colleges established and professorships endowed to follow up researches concerning the parasites that infest the limbs of these nineteenth century microbes. Science stands in danger of degenerating into a groping among the infinitely little things of darkness and evil, rather than walking in the infinitely great light of truth and purity. It is as though I saw a man trampling through a field of standing corn to bind up one broken plant and leaving behind him a trail of bruised and down-trodden heads of wheat. That is the tendency you scientists of to-day should avoid, so loading up your vessels of healing that their mission of usefulness may to the great mass of humanity mean an overpowering burden. Stand not above but among the people, justifying your existence as apostles of health, by a wiser way, and nearer sympathy with the heart of Nature and her simple, wholesome laws."

